# Breakfast and cognition: sixteen effects in nine populations, no single recipe

**DOI:** 10.3389/fnhum.2013.00631

**Published:** 2013-10-01

**Authors:** Tanya Zilberter, Eugene Y. Zilberter

**Affiliations:** ^1^Infotonic Conseil, Marseille, France; ^2^School of Psychology, University of Glasgow, Glasgow, UK

**Keywords:** cognitive performance, children, adults, metabolic stability, skipping breakfast, breakfast composition, intermittent fasting, intermittent ketosis

There is a consensus regarding the universal significance of breakfast (BF) for health, wellbeing, and cognition. The success of free school BF programs (e.g., Hasz and Lamport, 2012), which reportedly improve academic performance, fortifies this belief. However, studies showing cognitive effects of BF *vs*. skipping BF in large mixed cohorts of children (e.g., Wesnes et al., 2012) are often lacking metabolic and nutritional specifics. This creates uncertainty regarding the metabolic consequences of BF. Another uncertainty exists regarding skipping BF, which has been argued to have universally negative cognitive consequences—a claim that was recently announced a “*presumption*” unsupported by scientific evidence (Casazza et al., 2013, p.1). Surprisingly, in discussions regarding skipping BF, the neuroprotective and cognitively beneficial effects of intermittent fasting (IF) (featuring skipping BF every other day), although well-documented, are never mentioned.

Furthermore, the positive effect of free school BF on academic performance (previously unquestionable) was not supported in a randomized controlled trial (Mhurchu et al., 2013). In a meta-analytical review (Adolphus et al., 2013), behavioral results of school BF programs were considered lacking “*scientific rigor*.” Importantly, although it has been shown that the macronutrient content of BF “*can exert small but reliable effects independent of energy value and oro-sensory qualities*” (Lloyd et al., 1996, p. 1), this aspect is also unspecified in many of BF studies.

In this opinion article, we argue against the prevalent viewpoint of the universal benefits of BF by selectively highlighting issues demonstrating the complexity of the cognitive effects:

- The differences in cognitive effects of BF depending on age and baseline metabolic characteristics
- The specificity of BF composition
- The potential relationship of data on prolonged overnight fast (due to skipped BF) with data on the cognitive effects of IF and ketogenic diets (KD).

## Children

### Malnourished children

It is routinely stated that BF improves cognitive performance *especially* in malnourished children (e.g., Adolphus et al., 2013). This implies that other groups of children also benefit from BF, which does not seem to be the case. Omitting BF once in malnourished children worsened such cognitive outcomes as computational skills, problem solving, visual and auditory short-term memory, comprehension, and generation of ideas (Simeon and Grantham-McGregor, 1989; Hoyland et al., 2009). Noteworthy, cognitive ability and mental processing in malnourished (underweight) children was poorer, compared to controls, independently of BF (e.g., Bisset et al., 2012).

### Well-nourished children

Among 8–10 years old well-nourished children who regularly consumed BF, skipping it once did not affect any of the following cognitive performance tasks: visual motor function, executive function/spatial problem solving, psychomotor function/speed of processing, visual attention/vigilance, visual learning and memory, and attention/working memory (Kral et al., 2012). Pollitt et al., (1981), (1983) showed that in 9–11 years old well-nourished children, skipping BF actually decreased the number of errors in memory recall.

### Obese children

Obesity *per se* in 4–7 year olds did not impair cognitive abilities (Bisset et al., 2012). Skipping BF, however, resulted in a reduction of carbohydrate (CHO) utilization parallel to a decrease in attention (Maffeis et al., 2012). Improving the metabolic profiles of obese children (via therapy with Leptin) improved their verbal, non-verbal, and short-term memory (Paz-Filho et al., 2008).

### Children with different iqs

Not only nourishment but also children's intelligence influences the cognitive outcomes of skipping BF occasionally. Those with IQ above average (>100) increased the speed of information processing, which negatively correlated with blood glucose levels. Children with IQ below average had impaired cognitive performance as a result of skipping BF, with no correlation between glucose levels and performance (Pollitt et al., 1981).

## Brain structure and bf staple foods

The way a meal affects blood glucose (assessed by glycemic index, GI) influences cognitive consequences of BF. In children, 2 h after intake, low-GI BF has either less deteriorative effects (compared to high-GI BF) on accuracy of attention and secondary memory (Ingwersen et al., 2007) or improved declarative-verbal memory. On the other hand, high-GI BF resulted in better vigilance (Micha et al., 2012). Contrary to the effects of GI, glycemic load had no effects on cognition in 10–12-years-old children (Brindal et al., 2012).

Meticulous work by Taki et al., (2010) demonstrated amazing long-term effects of two nutritionally close BF staples (rice vs. bread) on children's brain morphology and one of the IQ components, the Perceptual Organization Index. This index was higher in the group regularly eating rice for BF, after adjusting for age, gender, socioeconomic family status, regularity of eating BF, and the variety of foods complementary to rice. Importantly, children in the rice-eating group had a significantly greater volume of gray matter. The Japanese variety of rice produces two times smaller disturbance in blood glucose compared to bread: GI of the Koshikari rice is 48, whereas the GI of bread is 100.

The gray matter volume correlation with cognition is further shown by Taki et al., (2012), where gray matter volume in the temporoparietal and prefrontal cortices positively correlated with full-scale (all components) IQ independently of meal composition, age, sex, and socioeconomic status. Similarly, reduction in volumes of gray matter (e.g., in the temporoparietal cortex) in adults is associated with mild cognitive impairment (Baron et al., 2001).

## Adults

### Breakfast composition

Generally speaking, “stable metabolic conditions seem to stabilize cognitive performance” (Fischer et al., 2002, p. 411) while “deviation from habitual meal composition can produce a relative decline in mood state” (Lloyd et al., 1996, p.1).

Studying short-term effects of BF in healthy young adults, Fischer et al., (2001) showed that the best cognitive performance occurred in habitual BF eaters after a morning meal of pure fat (butter), as opposed to isocaloric protein-rich or high-CHO meals. The fat meal provided the most constant metabolic condition, judged by the ratio of glucagon to insulin concentrations in the blood. On the other hand, a decreased tolerance to glucose has repeatedly shown to result in cognitive impairment (Grodstein et al., 2001; Hiltunen et al., 2001; Elias et al., 2005). (De Feo et al., 1988) showed that even a modest but consistent decrement in glucose stability caused an early impairment in cognitive function. Low-GI BF improved both memory test performance in humans and operant conditioning tasks in rats (Benton et al., 2003) but did not influence performance in an intelligence test (Benton and Parker, 1998).

### Glucose tolerance

Nabb and Benton, (2006) showed that in glucose-tolerant adults, a single high-CHO BF resulted in improvement in the Immediate Recall Memory Test. Glucose-intolerant adults, however, did not show any cognitive improvement. In both glucose-tolerant and intolerant subjects memory scores negatively correlated with BF calorie content. High-fat/high-CHO BF in this experiment caused information processing enhancement in those with high glucose tolerance while high-fat/low-CHO BF improved vigilance in those with low glucose tolerance.

### Skipping breakfast prolongs the overnight fast

Sleeping energy expenditure was higher when BF was habitually skipped indicating a prolongation of overnight ketosis (Kobayashi et al., in press). As mentioned above, the best cognitive performance was observed in habitual adult BF eaters after a BF of pure fat (Fischer et al., 2001), which may metabolically mimic the effects of skipping BF altogether by the same token as the KD mimics the effects of starvation (e.g., Beckett et al., 2013). Long-term effects of KD are known to be strongly neuroprotective (e.g., Zilberter, 2011) and cognitively beneficial, for instance in children (Hallböök et al., 2012) and in studies of Alzheimer's disease (Stafstrom and Rho, 2012; Beckett et al., 2013).

It is becoming evident that long-term effects of IF can be as efficient as continuous caloric restriction, which is well known for its beneficial metabolic and cognitive effects. Prolonging the overnight fast habitually happens every other day during standard IF or on a daily basis during time-restricted feeding (tRF). The standard version of IF prescribes a 24-h period of unrestricted eating followed by 24 h of caloric restriction (Johnson et al., 2006) or by complete fasting. In animal studies, tRF protocols restrict food availability to 4–8 h every day (e.g., Hatori et al., 2012). In humans, tRF is achieved by consistently reducing daily meal count and is considered more feasible than IF (Berardi et al., 2011). Animal studies have shown that metabolic consequences of tRF are similar to IF and are favorable independently of caloric intakes (Eshghinia and Mohammadzadeh, 2013). Even a short-term IF intervention in adult rats slowed age-associated decline in learning and improved cognitive functions (Singh et al., 2012). Anson et al., (2003) showed more pronounced effects lyof IF on glucose tolerance and insulin sensitivity compared to caloric restriction. Similarly, tRF has been shown to be as metabolically favorable in humans (Stote et al., 2007). In humans, IF showed long-term neuroprotective effects, e.g., in the prevention of neurodegenerative diseases (Love, 2005; Patel et al., 2005; Jadiya et al., 2011; Srivastava and Haigis, 2011), supposedly *via* improving synaptic plasticity and cognitive function (Araya et al., 2008; Fontán-Lozano et al., 2008; Liu et al., 2013).

It should be mentioned that in humans, during long-term as well short-term protocols, both IF and caloric restriction are hard to comply with due to persistent hunger (e.g., Stote et al., 2007). This difficulty is purely psychological in nature. In a within-subject experiment where two meals similar in taste and texture were administered, one containing calories and the other not (Lieberman et al., 2008), the authors concluded: “*Cognitive performance, activity, sleep, and mood are not adversely affected in healthy humans by 2 days of calorie deprivation when the subjects and investigators are unaware of the calorie content of the treatments*” (p. 667). Similar results were shown in sports medicine research: merely rinsing the mouth with CHO-containing drink without actually swallowing immediately enhanced exercise performance (Jeukendrup and Chambers, 2010).

### Intermittent ketosis

CHO restriction in high-fat diets induces chronic ketosis and mimics the metabolic consequences of fasting (Barañano and Hartman, 2008; Zilberter, 2011; Stafstrom and Rho, 2012). By the same token, a high-fat/low-CHO BF mimics the metabolic features of IF or tRF. Eating a very high-fat BF, as mentioned above, improved cognition (Fischer et al., 2001), which may be due to prolongation of the overnight fast. (Freemantle et al., 2009) showed that a ketogenic BF does not interrupt the overnight ketosis. Consequently, both the “ham and egg” style BF (Smith et al., 1994) and skipping BF result in the metabolic condition that can be defined as intermittent ketosis (IK). IK occurs, for example, in followers of the Carbohydrate Addict Diet (Heller and Heller, 1994) allowing CHO intake only once a day, along with any amount of additional meals containing little or no CHO (essentially ketogenic). When this diet is mentioned in peer-reviewed publications (very seldom), it is never distinguished from other low-CHO diets despite having a significant advantage due to it's potential of combining the benefits from both low-CHO diets and IF/tRF.

Although Heller and Heller, (1994) did not mention BF as a ketogenic meal, it is logical to suppose that prolonging overnight ketosis by high-fat/low-CHO BF supports IK during CAD. The matter is, CHO cravings (an element of CHO addiction) are thought to correspond to afternoon/evening drops in brain serotonin levels causing dysphoria as well as other cognitive effects of serotonin depletion (Spring et al., 2008). This can explain why successful CAD dieters prefer to have their CHO-rich meals in the evening although the diet does not prescribe an exact time for it.

## Conclusion

**Figure 1:**
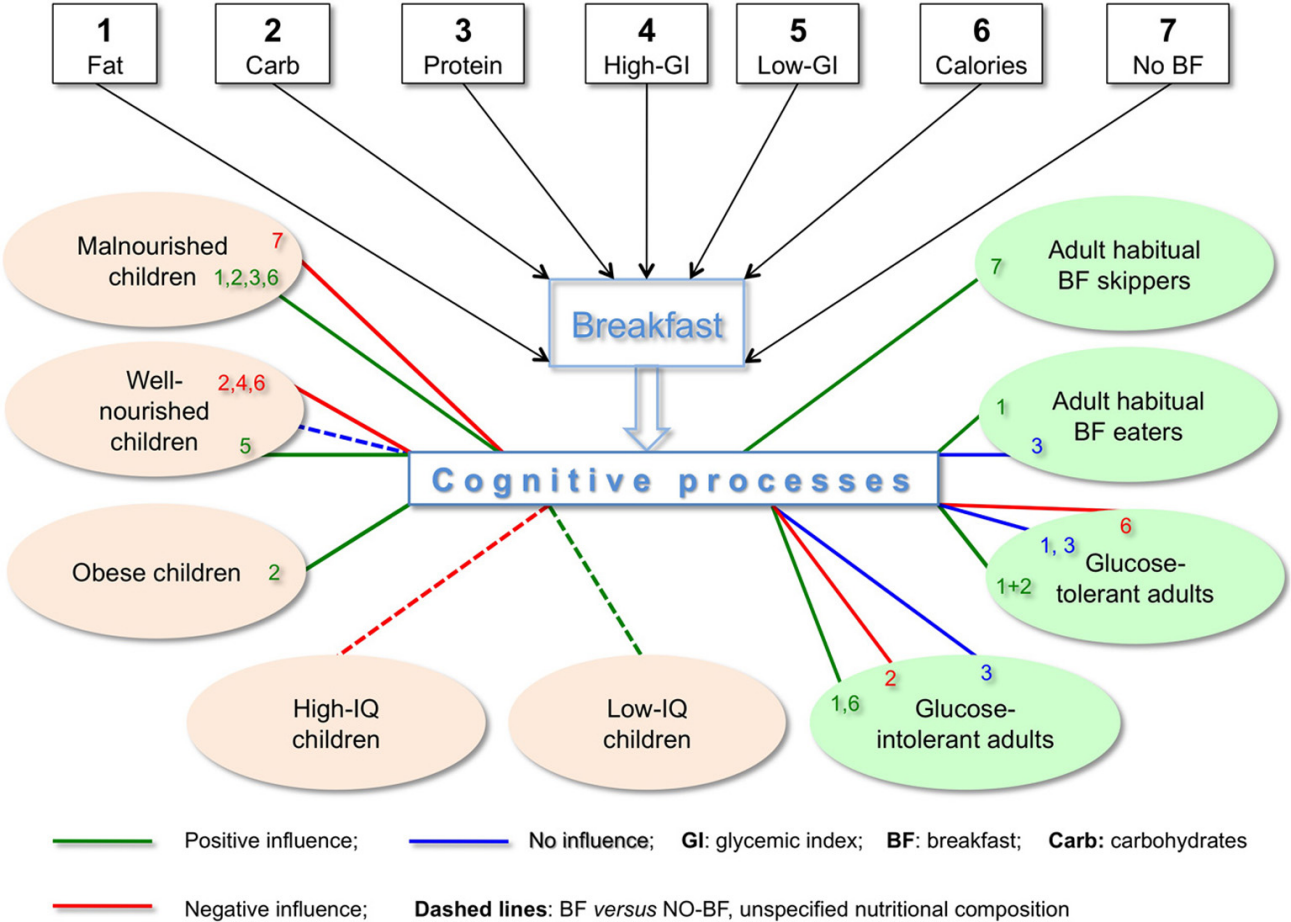
**Cognitive effects depend on nutritional composition of breakfast and characteristics of subjects.** Effects on cognition in children depend on BF composition (macronutrients, GI, calories), nourishment (malnourished, well-nourished, obese) and IQ. In adults, cognitive effects of BF also depend on BF composition as well as on glucose tolerance and whether or not they eat BF habitually. Contrary to the broadly accepted belief, high-CHO BF may negatively influence cognition in well-nourished children. Also quite contrary to the common view of healthy eating, high-fat BF improved cognitive performance in adult habitual BF eaters. Chronic and/or acute conditions, wherever the information is available, are specified in the text.

The complexity of the results described in this opinion article is depicted in Figure 1, where we see that seven metabolically distinct BF types have 16 different effects on nine populations of children and adults, including direct data on positive cognitive effects of skipping BF (e.g., on immediate recall in short-term memory, Pollitt et al., 1981, 1983) as well as positive metabolic and/or cognitive effects shown in IF and tRF protocols featuring skipping BF (Love, 2005; Patel et al., 2005; Jadiya et al., 2011; Srivastava and Haigis, 2011; Singh et al., 2012). As succinctly summarized by Bellisle (2004, p. S230), skipping BF “*has deleterious effects, has no effect or even has beneficial effects depending on what the task is, when it is performed after breakfast, the child*'*s IQ, the child*'*s age and nutritional status*.” Clearly there is no single recipe for BF, and the statement “*Breakfast is the most important meal of the day*” is not as unequivocal as it is widely thought to be.
